# Disruption of Glucocorticoid Action on CD11c^+^ Dendritic Cells Favors the Generation of CD4^+^ Regulatory T Cells and Improves Fetal Development in Mice

**DOI:** 10.3389/fimmu.2021.729742

**Published:** 2021-10-26

**Authors:** Lianghui Diao, Alexandra Maximiliane Hierweger, Agnes Wieczorek, Petra Clara Arck, Kristin Thiele

**Affiliations:** Division of Experimental Feto-Maternal Medicine, Department of Obstetrics and Fetal Medicine, University Medical Center Hamburg-Eppendorf, Hamburg, Germany

**Keywords:** dendritic cells, glucocorticoids, placenta, progesterone, regulatory T cells (T reg)

## Abstract

A wealth of innate and adaptive immune cells and hormones are involved in mounting tolerance towards the fetus, a key aspect of successful reproduction. We could recently show that the specific cross talk between the pregnancy hormone progesterone and dendritic cells (DCs) is significantly engaged in the generation of CD4^+^ FoxP3^+^ regulatory T (Treg) cells while a disruption led to placental alterations and intra-uterine growth restriction. Apart from progesterone, also glucocorticoids affect immune cell functions. However, their functional relevance in the context of pregnancy still needs clarification. We developed a mouse line with a selective knockout of the glucocorticoid receptor (GR) on DCs, utilizing the cre/flox system. Reproductive outcome and maternal immune and endocrine adaptation of Balb/c-mated C57Bl/6 GR^flox/flox^CD11c^cre/wt^ (mutant) females was assessed on gestation days (gd) 13.5 and 18.5. Balb/c-mated C57Bl/6 GR^wt/wt^CD11c^cre/wt^ (wt) females served as controls. The number of implantation and fetal loss rate did not differ between groups. However, we identified a significant increase in fetal weight in fetuses from mutant dams. While the frequencies of CD11c^+^ cells remained largely similar, a decreased expression of co-stimulatory molecules was observed on DCs of mutant females on gd 13.5, along with higher frequencies of CD4^+^ and CD8^+^ Treg cells. Histomorphological and gene expression analysis revealed an increased placental volume and an improved functional placental capacity in mice lacking the GR on CD11c^+^ DCs. In summary, we here demonstrate that the disrupted communication between GCs and DCs favors a tolerant immune microenvironment and improves placental function and fetal development.

## Introduction

Over the course of gestation, the maternal immune system needs to adapt to the semi-allogenic fetus in order to prevent fetal rejection and promote successful pregnancy maintenance. Both, the innate and adaptive immune system are involved in a synergistic interplay of multiple processes occurring at the fetal-maternal interphase. Essentially hereby is the arrest of dendritic cells (DC) in a tolerogenic state (1, 2), the restricted migration of effector T cells to the feto-maternal interface along with the generation of Treg cells (2, 3). These processes are modulated by pregnancy hormones such as progesterone, glucocorticoids (GCs) and estradiol (4, 5). Although the significance of such hormone-immune cell communication is well established, specific insights on how hormones such as steroids modulate distinct cell subsets are still missing.

By using the cre/lox system, we have recently shown that the specific cross talk between progesterone and DC acts as a decisive factor for the establishment of maternal immune tolerance. Disruption of this cross talk resulted in an impaired maternal immune adaptation, reflected by a decline of tolerogenic DCs and decreased frequencies of CD4^+^ and CD8^+^ Treg cells in the uterus. These immune changes were accompanied by altered histomorphological features of the placenta and reduced fetal growth and development, which was independent of fetal sex and genotype (6).

Besides progesterone, other hormones, such as estradiol, human chorionic gonadotropin (hCG) and GCs, are known to affect immune cell functions (7). The latter is also increasing over the course of pregnancy, peaking at parturition (8). GCs acts *via* the intracellular glucocorticoid receptor (GR), which is expressed in almost every cell (9, 10). However, the functional role of GCs in the context of pregnancy and the establishment of immune tolerance to the fetus has not been comprehensively assessed.

Therefore, we established a mouse model, which allows assessing the impact of GCs in modulating DC function during pregnancy by generating a cell-specific knockout of the GR on DCs.

## Materials And Methods

### Generation of GR^flox/flox^CD11c^cre/wt^ Mice

**Figure 1:**
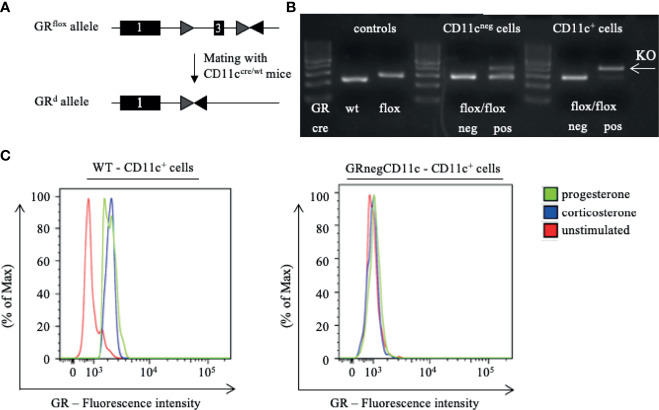
Generation of a selective knockout (KO) of the glucocorticoid receptor (GR) on CD11c dendritic cells (DCs). **(A)** GR^flox/flox^ mice were crossed with CD11c^cre/wt^ transgenic mice resulting in recombination of the GRflox allele to a GR null allele (GRd) on CD11c^+^ cells. **(B)** The selective KO of the GR on CD11c^+^ cells was confirmed on DNA level. PCR amplicons were visualized using agarose gel electrophoresis and band sizes were 225 bp for the wildtype allele, 275 bp for the floxed allele, and 390 bp for the KO allele, respectively. **(C)** The selective KO of the GR on CD11c^+^ DCs harvested from WT and GRnegCD11c mice was confirmed on protein level by means of flow cytometry. Cells were preincubated with either progesterone or corticosterone to enable GR staining. Fluorescence intensity histogram confirms the presence (left) or absence (right) of the GR, respectively.

GR^flox/flox^ mice (JAX stock #021021) carrying loxP sites flanking exon 3 of the nuclear receptor subfamily 3, group C, member 1 (Nr3c1) gene (11) and CD11c^cre/wt^ mice (JAX stock #008068) expressing a cre recombinase under control of the integrin alpha X gene (Itgax or Cd11c) promoter region (12) were kindly provided by Manuel Friese (University Medical Center Hamburg-Eppendorf, Hamburg, Germany). In order to generate female mice lacking the GR on CD11c^+^ dendritic cells, GR^flox/flox^ females and CD11c^cre/wt^ males were mated in the Animal Facility of the University Medical Center Hamburg-Eppendorf (**Figure 1A**). Subsequently, male GR^flox/wt^ CD11c^cre/wt^ and female GR^flox/wt^ CD11c^wt/wt^ offspring were mated in order to generate GR^flox/flox^ CD11c^cre/wt^ and GR^wt/wt^ CD11c^cre/wt^ animals, respectively. Cre expression was always transmitted from the father in order to avoid a premature impact of an impaired GC-DC-cross talk. Hence, GR^wt/wt^CD11c^cre/wt^ were obtained by mating male PR^wt/wt^ CD11c^cre/wt^ with PR^wt/wt^ CD11c^wt/wt^ females. For simplicity we will refer to them as WT mice. Mice with the conditional KO were maintained by mating GR^flox/flox^ CD11c^cre/wt^ males with GR^flox/flox^ CD11c^wt/wt^ female and we will refer to them as GR^neg^CD11c.

We are aware that the cre-lox system exhibit certain limitations such as the efficiency of target gene deletion, cre-mediated toxicity and undesired deletions (13). The latter two were avoided by using GR^wt/wt^CD11c^cre/wt^ mice as controls. Consequently, both groups share the same potential cre-mediated toxicity burden.

### Isolation of CD11c^+^ and CD11^neg^ Cells

For the confirmation of the selective KO of the GR on CD11c dendritic cells, we harvested the spleen from WT and GR^neg^CD11c mice. Single cell suspensions were obtained as described previously (6). In brief: the tissue was mashed with the plunger of a sterile disposable syringe in circles through a 40 µm cell strainer (Falcon Cell Strainer 40 μm, BD Bioscience, VWR, Germany). The resulting cell suspension was centrifuged for 8 minutes with 450 x g at 4°C and the supernatant was discarded. A red cell blood (RBC) lysis was performed using RBC lysis buffer (eBioscience, San Diego, CA) according to the manufacturer’s instruction. After centrifugation the cell pellet was resuspended in PBS. CD11c^+^ and CD11^neg^ cells were sorted using a FACS AriaFusion (BD Biosciences) to achieve highest purity.

### DNA Isolation and Polymerase Chain Reaction (PCR)

DNA was isolated from mouse tail and CD11c^+^ and CD11c^neg^ cells obtained from WT and GR^neg^CD11c mice using the DNeasy Kit (Qiagen) according to the manufacturer’s protocol. PCR analysis was performed as 3-Primer-PCR in 50 μl reactions using the Mastercycler^®^ nexus GX2 (Eppendorf). The following primer sequences 5’-GGCATGCACATTACTGGCCTTCT-3’, 5’-GTGTAGCAGCCAGCTTACAGGA-3’ and 5’-CCTTCTCATTCCATGTCAGCATGT-3’ were ordered from TIB Molbiol. The PCR program consisted of initial 94°C for 10 min followed by 30 cycles: 94°C for 1 min, 55°C for 1 min, and 72°C for 2 min. Amplicons were visualized using Agarose gel electrophoresis. Expected band sizes were 225bp for the wildtype allele, 275 bp for the floxed allele and 390 bp for the KO allele.

### Timed Pregnancies

Eight to ten week old GR^neg^CD11c female mice with a C57Bl/6 background were allogenically mated overnight to Balb/c male mice. Aged-matched Balb/c-mated WT females served as controls. The presence of a vaginal plug in the morning was considered as gestation day (gd) 0.5. Maternal weight was controlled on gd 8.5 and 10.5 to confirm ongoing pregnancy. Animals were kept under 12 h light/dark cycles and received food and water ad libitum. All experiments were carried out in accordance with the animal ethics approval given by the State Authority of Hamburg (ORG_952).

### Tissue Harvesting

Mice were anesthetized with CO_2_/O_2_ on gd 13.5 or 18.5, respectively. A blood sample was collected by retro bulbar puncture and subsequently mice were sacrificed by cervical dislocation. The harvested uterus-draining lymph node was kept in PBS on ice. The fetuses and corresponding placentas were isolated from the amniotic membranes. After assessment of fetal weight, fetuses were fixed with Bouin’s solution. The empty uterus was stored in HBSS on ice. Placentas were either stored at -20°C in RNAlater (Ambion by Life Technologies GmbH) for subsequent gene expression analysis or embedded in biopsy cassettes and stored in 4% Formaldehyde solution (36.5-38%, Sigma-Aldrich, St, Louis, US) for 24 h before transfer into 1% Formaldehyde solution for long-term storage and histological staining.

### Pregnancy Outcome

Number of implantations and abortions were counted per pregnant female. The abortion rate was calculated by the following equation: (number of abortions/number of implantations) * 100.

### Tissue Processing

Single cell suspensions of maternal lymph nodes and uteri were obtained as described before (6, 14). In brief, maternal lymph nodes were passed through a cell strainer and after centrifugation at 450 g for 8 minutes at 4°C, the cell pellet was resuspended in PBS. The uterus was enzymatically digested using 200 U/mL hyaluronidase (Sigma-Aldrich), 1 mg/mL collagenase VIII type (Sigma-Aldrich), and 1 mg/mL bovine serum albumin fraction V (Sigma-Aldrich) dissolved in 5 mL HBSS. Subsequently, the uterus was incubated twice for 20 minutes in a 37°C water bath with agitation. Intermediately, the solution was recovered and filtered through a mesh. The solution was centrifuged at 450 x g for 8 minutes at 4°C and resuspended PBS. PBMCs were isolated from blood samples by using 1x Red Blood Cell (RBC) lysis buffer (eBioscience, Invitrogen by Thermo Fisher Scientific) according to the manufacturer’s instructions. Lysis was stopped with PBS and subsequently, samples were centrifuged at 450 x g for 8 minutes at 4°C and resuspended PBS.

Number of viable leukocytes in all tissues was obtained by counting the cells using a Neubauer chamber upon adding Trypan Blue stain (0.4%, Life Technologies GmbH, Darmstadt, Germany).

### Flow Cytometry

For flow cytometric analyses, 1.0x10^6^ maternal lymph node and uterus cells and 0.5x10^6^ PBMCs were used for immuno-phenotyping. Non-specific binding was blocked by rat anti-mouse CD16/CD32 Mouse Fragment crystallizable (Fc) Block (1:200, BD Bioscience) and Normal Rat Serum (1:100, eBioscience) for 15 min at 4°C. Subsequently, the cells were incubated with the respective antibodies for 30 min for surface and intracellular staining. Antibodies are summarized in **Table 1**. In order to identify dead cells, cells were simultaneously stained with eFluor 506 viability dye (eBioscience). For intracellular staining, cells were fixed and permeabilized using Foxp3 Fixation/Permeabilization Concentrate and Diluent (eBioscience) according to the manufacturer’s instructions.

**Table 1:** Summary of antibodies used in the present study.

Target antigen	Fluorochrome	Clone	Dilution	Source
anti-CD45	Allophycocyanin (APC)-Cyanine (Cy)7	30-F11	1:400	BD
anti-CD3	R-phycoerythrin (PE) Cy7	145-2C11	1:200	Biolegend
anti-CD8	Brilliant Violet (BV) 650	53-6.7	1:100	Biolegend
anti-CD4	Pacific Blue	RM4-5	1:400	Biolegend
anti-FoxP3	PE	FJK-16s	1:200	eBioscience
anti-CD122	PerCP eFluor 710	TM-b1	1:100	eBioscience
F4/80	BV421	BM8	1:100	Biolegend
anti-CD11c	BV785	N418	1:100	Biolegend
MHCII	APC	M5/144.15.2	1:200	BD
anti-CD80	BV605	16-10A1	1:100	Biolegend
anti-CD86	BV605	GL-1	1:100	Biolegend

In order to quantify the expression of the GR by flow cytometry, 2x10^6^ cells were first incubated with 10^-6^ M progesterone or corticosterone or only medium for 15 minutes, respectively. Subsequently, samples were blocked and stained with the respective surface markers for 30 minutes. Afterwards, cells were fixed and permeabilized as stated above and intracellularly stained with an anti-GR antibody directly labelled with AF488 for 30 minutes.

Flow cytometric data were acquired using a BD LSRFortessa II (BD Biosciences) and analyzed using FlowJo (Tree Star, Ashland, OR, USA).

### Placental Histology

Paraffin embedded placentas were cut into 4 µm thick histological sections at the mid-sagittal plane using a microtome (SM2010R, Leica, Bensheim, Germany). Slides were dewaxed and rehydrated using xylene and ethanol. Masson-Goldner trichrome staining was performed following standard protocol (15). Subsequently, slides were scanned with a Mirax Midi Slide Scanner. Histomorphological analyses of placental areas were performed by two independent observers using Panoramic Viewer (3DHistech Kft. Budapest, Hungary).

### Progesterone Analysis

Maternal blood samples were centrifuged at 10.000 g for 20 min at 4°C and the supernatant plasma was immediately frozen at -20°C. For progesterone analysis, plasma samples were diluted 1:200 using ELISA Buffer and measured with a competitive immunoassay (Progesterone ELISA Kit, Cayman Chemical, Michigan, USA) on a NanoQuant (Tecan Group AG, Männedorf, Switzerland) according to manufacturer’s instructions.

### Theiler Scoring of Fetuses on gd13.5

Fetal development of mouse embryos was determined by investigation of Bouin-fixed fetuses under a Zeiss Stemi 2000-C stereomicroscope according to Theiler’s description (16). Main criteria to differentiate the developmental stages at gd 13.5 have been the formation of the pinna, fingers and feet, and the presence or absence of 5 rows of whiskers.

### RNA Isolation and cDNA Synthesis

RNA from placenta tissue was isolated as described previously (6). Briefly, tissue was homogenized using micro packaging vials with ceramic beads (1.4 mm) in a Precellys^®^ 24 Tissue Homogenizer (Peqlab). RNA isolation and DNA digestion were conducted by utilizing the RNeasy Plus Universal Mini Kit (QIAGEN) and DNA-free Kit (Applied Biosystems by Thermo Fisher) respectively. cDNA synthesis was conducted with random primers (Invitrogen by Thermo Fisher). Concentration and purity of RNA and cDNA were assessed employing the Multiskan SkyHigh Microplate Spectrophotometer (ThermoFisher Scientific).

### Quantitative Real-Time Polymerase Chain Reaction (qRT-PCR)

Gene expression analyses of placental tissue were carried out using gene expression assays (Applied Biosystems by Thermo Fisher) for the following targets: insulin like growth factor 1 (*Igf1*, Mm00439560_m1), hydroxysteroid 11-beta dehydrogenase (*Hsd11b*) 1 and 2 (Mm00476182_m1 and Mm01251104_m1), placental growth factor (*Pgf*, Mm00435613_m1), epidermal growth factor (*Egf*, Mm00438696_m1), vascular endothelial growth factor A (*Vegf*, Mm00437306_m1), B cell leukemia/lymphoma 2 (*Bcl2*, Mm00477631_m1) and soluble FMS-like tyrosine kinase 1 (*sFlt1*, Mm00438980_m1), heme oxygenase 1 (*Hmox1*, Mm00516005_m1), galectin-1 (*Gal-1*, Mm00839408_g1), and placental lactogen II (*Prl3b1*, Mm00435852_m1). RNA polymerase II subunit A (*Polr2a*, Mm00839502_m1) and ubiquitin C (*Ubc*, Mm02525934_g1) served as endogenous controls (17). All reactions were performed in 50 cycles using a standard two-step RT-PCR: initial 50°C for 2 min and 95°C for 10 min, 15 s denaturation at 95°C and 60 s annealing and extension at 60°C with the NanoQuant5 Real-Time PCR System (Applied Biosystems) and the corresponding software. The fold change of GRnegCD11c over WT control expression was calculated using the ΔΔCt method (18).

### Statistical Analysis

Statistical analysis was performed using GraphPad Prism version 7.0 (GraphPad Software, La Jolla, CA, USA). All results are expressed as means ± standard error of the mean (SEM). Means between groups were compared using Student’s t-test, after confirming Gaussian distribution.

## Results

### Confirmation of the Selective Knockout of the GR on CD11c^+^ DCs

First, we confirmed the selective knockout of the GR on CD11c^+^ DCs on DNA level. Therefore, we performed genotyping of tail biopsies and CD11c^+^ and CD11neg cells of WT and GRnegCD11c mice, respectively. The expected band size of amplicons from WT animals is 225 bp, a size of 275 bp was expected for the GRnegCD11c mice and could be confirmed (**Figure 1B** left). Subsequently, CD11c^+^ and CD11c^neg^ cells were sorted from GR^flox/flox^ animals with and without cre expression. PCR-based DNA segment amplification of sorted CD11c^neg^ spleen cells from these animals resulted in the 275 bp amplicon. CD11c^neg^ spleen cells from mice with the conditional knockout exhibit additionally a slight band at 390 bp, the expected band size of the KO allele, suggesting minor unspecificity. In contrast, PCR of sorted CD11c^+^ spleen cells from GRnegCD11c mice resulted in a 390 bp amplicon resembling the KO allele while the band for the floxed allele was mostly gone. These analyses confirmed the selective KO of the GR on DCs on a molecular level.

In order to confirm this selective KO also on the protein level, we used a monoclonal antibody against the GR for detection by flow cytometry. Splenic cells from WT and GR^neg^CD11c mice either remained unstimulated or were stimulated with progesterone and corticosterone, respectively, in order to ensure binding to the GR which was necessary for subsequent staining (19). Flow cytometry analysis demonstrated the presence of the GR on CD11c^+^ DCs derived from WT animals after stimulation with progesterone and corticosterone in comparison to unstimulated cells (**Figure 1C**). In contrast, CD11c^+^ DCs originating from GRnegCD11c mice failed to generate a positive GR signal confirming the selective KO (**Figure 1C**).

### Selective Knockout of the GR on CD11c^+^ DCs Results in Improved Fetal Outcome

**Figure 2:**
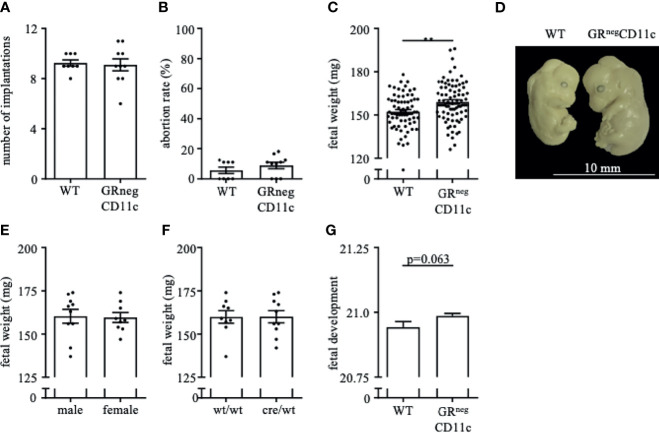
Selective knockout of the GR on CD11c^+^ dendritic cells results in improved fetal outcome. WT and GRnegCD11c female mice were allogenically mated to Balb/c males and pregnancy outcome was assessed on gestational day (gd) 13.5, maternal phenotype is always indicated below: number implantation **(A)**, abortion rate **(B)** and fetal weight **(C)**. A representative picture from gd 13.5 fetuses of WT (left) and GRnegCD11c (right) mice is shown in **(D)**, white line in the picture denotes 10mm. Fetal weight of fetuses from GRnegCD11c dams is further shown dependent on fetal sex **(E)** and fetal genotype **(F)**. **(G)** Fetal development was scored on gd13.5 according to Theiler criteria. Scatter-bar-plots represent mean ± SEM, Student’s *t* test, **p ≤ 0.01. Each dot in the scatter plot represents a single animal.

Pregnancy outcome assessed on gd 13.5 showed no differences between WT and GR^neg^CD11c dams with regards to the number of implantation (**Figure 2A**) and fetal loss rate (**Figure 2B**). However, fetal weight was higher in offspring derived from GR^neg^CD11c compared to WT mothers (**Figure 2C**). Representative photographs of fetuses from WT and GRnegCD11c female mice are shown in **Figure 2D**. The observed change in fetal weight was not dependent on fetal sex (**Figure 2E**) nor on fetal genotype, which is different between litter mates due to the heterozygosity of the cre (**Figure 2F**). Additionally, Theiler staging revealed a slightly advanced fetal development in GRnegCD11c dams (**Figure 2G**).

### Impaired Glucocorticoid-Responsiveness of CD11c^+^ DCs Improves Placenta Function

**Figure 3:**
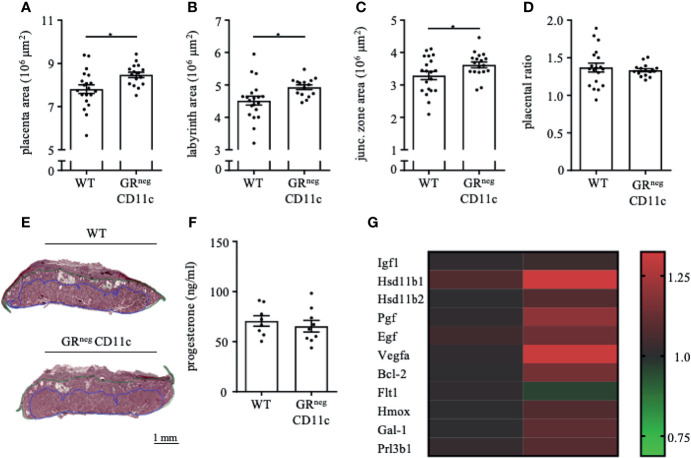
Impaired Glucocorticoid-Responsiveness of CD11c^+^ dendritic cells improves placenta function. WT and GRnegCD11c female mice were allogenically mated and placentas harvested on gestation day (gd) 13.5 were evaluated by Masson-Goldner trichrome staining allowing the differentiation of the labyrinth and junctional zone. Total placenta area **(A)**, area of the labyrinth **(B)** and the junctional zone **(C)** have been assessed and the placental ratio (D, labyrinth/junctional zone) was calculated. **(E)** Representative photomicrographs illustrating mid-sagittal sections of gd 13.5 placental tissue from WT (top) and GRnegCD11c (bottom) mothers, black line in the picture denotes 1mm, blue lines encircle the labyrinth, green lines surround the junctional zone. **(F)** Plasma progesterone levels of WT and GRnegCD11c female mice on gd 13.5 as analyzed by ELISA. Scatter-bar-plots represent mean ± SEM, Student’s t test, *p ≤ 0.05. Each dot in the scatter plot represents a single placenta **(A–D)** or a single animal **(F)**, respectively. **(G)** Heatmap summarizing the placental expression of insulin like growth factor 1 (Igf1), hydroxysteroid 11-beta dehydrogenase 1 and (Hsd11b1 and 2), placental growth factor (Pgf), epidermal growth factor (Egf), vascular endothelial growth factor A (Vegfa), B cell leukemia/lymphoma 2 (Bcl2), soluble FMS-like tyrosine kinase 1 (sFlt1), heme oxygenase 1 (Hmox1), Galectin-1 (Gal-1), and placental lactogen II (Prl3b1) calculated by qPCR from gd 13.5 placentas. The fold change over WT control expression was calculated using RNA polymerase II subunit A (Polr2a) and ubiquitin C (Ubc) as endogenous control and employing the δδCt method.

In order to assess the placental ratio (labyrinth/junctional zone) as a proxy for placental function, we performed Masson-Goldner trichrome staining on mid-sagittal sections on gd 13.5. We could observe that the overall placenta surface area was significantly increased in GR^neg^CD11c females compared to WT controls (**Figure 3A**). This increase was equally distributed between labyrinth and junctional zone, because both areas were detected to be enlarged also individually (**Figures 3B, C**). Consequently, the placental ratio did not differ between groups (**Figure 3D**). Representative photomicrographs from gd 13.5 placentas are shown in **Figure 3E**. These placental changes were not accompanied by changes in serum progesterone levels (**Figure 3F**).

We further analyzed the differential expression of placental genes that have been linked to placental function (**Figure 3G**). Here, we could demonstrate that placental growth factor (*Pgf*), vascular endothelial growth factor A (*Vegfa*) and B cell leukemia/lymphoma 2 (*Bcl-2*) were significantly increased in placentas from GRnegCD11c mothers. In addition, hydroxysteroid 11-beta dehydrogenase 1 and 2 (*Hsd11b1* and *2*), heme oxygenase 1 (*Hmox*), and galectin-1 (*Gal-1*) showed a trend toward increased expression in GRnegCD11c placentas, but did not reach levels of significance. Insulin like growth factor 1 (*Igf1)*, epidermal growth factor (*Egf*), soluble FMS-like tyrosine kinase 1 (*sFlt1*), and placental lactogen II (*Prl3b1*) were not affected by the selective KO of the GR on DCs.

### Impaired Glucocorticoid-Responsiveness of CD11c^+^ DCs Mitigates a Pregnancy-Protective Immune Environment

**Figure 4:**
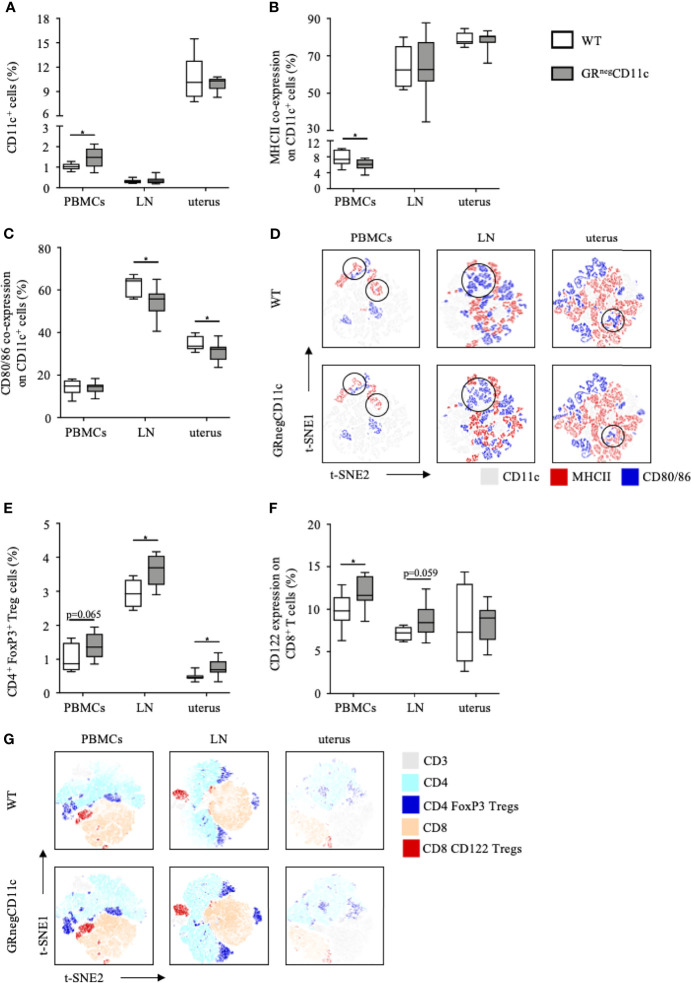
Impaired glucocorticoid-responsiveness of dendritic cells (DCs) beneficially affects maternal immune adaption. WT and GRnegCD11c female mice were allogenically mated and flow cytometric analysis of peripheral blood mononuclear cells (PBMCs), the uterus-draining lymph node (LN) and the uterus was performed on gestation day 13.5. Box plots present the frequencies of **(A)** CD11c^+^ DCs, **(B)** the co-expression of the major histocompatibility complex (MHC) II and **(C)** CD80 and CD86. Representative tSNE plots are shown in **(D)** Box plots present the frequencies of CD4^+^FoxP3^+^ Treg cells **(E)** and CD122^+^ expression in CD8^+^ T cells **(F)** of WT and GRnegCD11c mice. Representative TSNE are shown in **(G)** Box plots represent mean ± SEM, Student’s t test, *p ≤ 0.05, unless otherwise stated, cell frequencies are expressed as percentage of living CD45^+^ cells.

Flow cytometry analyses were performed for PBMCs, the uterus-draining lymph nodes and leukocytes isolated from the uterus on gd 13.5. Frequencies of CD11c^+^ DCs are rather low in PBMCs and the uterus-draining lymph nodes. However, we did observe higher frequencies of CD11c^+^ DCs among PBMCs in GRnegCD11c dams compared to WT, which was not present in the uterus-draining lymph nodes. In comparison, CD11c^+^ DCs are more abundant in the uterus, but similar frequencies were detected in both groups (**Figure 4A**). The expression of co-stimulatory molecules were found to be differentially modulated, with an immature, tolerogenic phenotype of CD11c^+^ DCs more frequently present in cells derived from GR^neg^CD11c dams. MHCII co-expression was significantly decreased in PBMCs, while no changes could be detected in the uterus and the draining lymph node (**Figure 4B**). In contrast, the co-expression of CD80/86 was similar in PBMCs, but significantly reduced in the draining lymph node and the uterus itself (**Figure 4C**). Representative t-SNE plots are shown in **Figures 4D**.

As immature, tolerogenic DCs are involved in the generation and activation of CD4 Treg cells, we further analysed the frequencies of this cell subset and identified increased frequencies of CD4^+^ FoxP3^+^ Treg cells among PBMC, uterus-draining lymph nodes and in uterus of GRnegCD11c dams compared to WT animals. However, levels of significance were not reached in PBMCs (**Figure 4E**). In addition, we could also detect increased CD122^+^ expression on CD8 T cells in PBMCs and the uterus-draining lymph node of in GRnegCD11c mothers, but not in the uterus (**Figure 4F**). Representative t-SNE plots are shown in **Figure 4G**.

### Improved Fetal and Placental Outcomes Remains Till the End of Pregnancy

**Figure 5:**
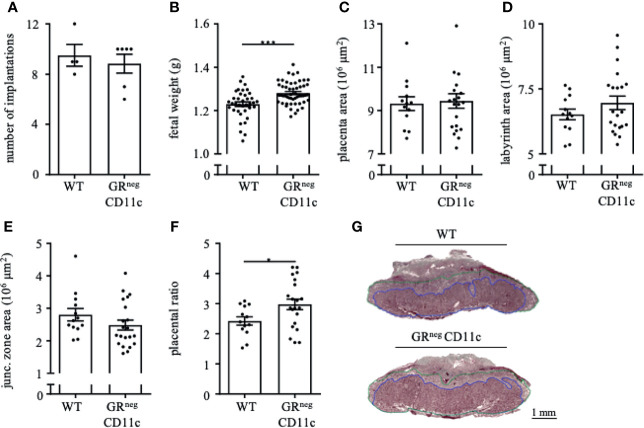
Improved fetal and placental outcome due to an impaired glucocorticoid-responsiveness of CD11c^+^ dendritic remains till the end of pregnancy. WT and GRnegCD11c female mice were allogenically mated to Balb/c males and pregnancy outcome was assessed on gestational day (gd) 18.5: number implantation **(A)**, fetal weight **(B)** and overall placental area **(C)**. Area of the labyrinth **(D)** and the junctional zone **(E)** have been assessed and the placental ratio **(F)** was calculated. **(G)** Representative photomicrographs illustrating mid-sagittal sections of gd 18.5 placental tissue from WT (top) and GRnegCD11c (bottom) mothers, black line in the picture denotes 1mm, blue lines encircle the labyrinth, green lines surround the junctional zone. Scatter-bar-plots represent mean ± SEM, Student’s *t* test, *p ≤ 0.05, ***p ≤ 0.001. Each dot in the scatter plot represents a single animal **(A, B)** or a single placenta **(C–F)**, respectively.

In order to evaluate if the observed immune and placental alterations are carried through until the end of pregnancy, we also assessed the reproductive outcome on gd 18.5. Similar to gd 13.5, we did not observe changes in implantation rate (**Figure 5A**) and abortion rate (data nor shown) between GRnegCD11c and WT dams. However, fetal weight was again significantly higher in offspring from GRnegCD11c mother compared to WT dams (**Figure 5B**). Opposed to the observations on gd 13.5, placental morphology showed no differences in the overall placental surface area on gd 18.5 in GRnegCD11c and WT dams (**Figures 5C**). However, we did observe a skew toward an increased labyrinth at the expense of the junctional zone in GRnegCD11c females, compared to WT females (**Figures 5D, E**) resulting in a significantly increased placental ratio (**Figure 5F**) (20). Representative photomicrographs from gd 18.5 placentas are shown in **Figure 5G**.

## Discussion

In the context of reproduction, maternal GCs are well known for their crucial role accommodating the maternal energy demand associated with pregnancy (21). GC also promote fetal lung maturation, which mitigates neonatal survival after birth. The latter has been observed in knockout mice, where offspring lacking the GR showed a severe lung phenotype and a 100% mortality at birth or shortly afterwards (22, 23). Consequently, GR knockout mice were identified not to be a suitable research tool. Hence, the availability of GR^flox/flox^ mice became essential to investigate GC-dependent pathways in highly target-specific approaches (11). However, investigations of cell- or tissue-specific function of the GR in the reproductive system is still very limited. To date, GR^flox/flox^ mice had been used to selectively deplete the GR in the uterus, which led to subfertility due to defects in implantation-related to inadequate remodeling of the endometrial stroma (24).

DCs have been shown to play a crucial role for pregnancy success as depletion of uterine DCs was associated with impaired decidual proliferation and differentiation as well as with perturbed angiogenesis leading to fetal resorptions (25). An impaired progesterone-DC cross talk is associated with intra-uterine growth restrictions, while implantation remains unaffected (6). Conversely, the disruption of GC action on DCs resulted in an improved fetal growth and placental formation, as shown in our present study.

Similar to progesterone, GCs can exert strong immuno-modulatory functions, ranging from pro- to anti-inflammatory effects (26). It is argued that GC-mediated pro-inflammatory activity is essential to respond sufficiently to pathogens (27) and that GC deficiency is associated with a defective immune response and recurrent infections (28). However, preliminary results on H1N1 influenza virus infection of pregnant GR^neg^CD11c mice did not indicate an increased morbidity (data not shown). The anti-inflammatory effects of GCs are generally more widely accepted. These effects include the induction of apoptosis of T and B cells, mature dendritic cells, basophils, and eosinophils (29) or the re-establishment of tissue homeostasis after inflammatory processes (30) by repressing pro-inflammatory genes encoding cytokines, chemokines, cell adhesion molecules and inflammatory enzymes (26). Further, GCs are reported to drive macrophages toward the M2 phenotype (31) and to prevent the differentiation and maturation of DCs (32). In our study, also mice lacking the GR on DCs exhibit a reduced expression of maturation marker and co-stimulatory molecules on DCs. These contradictory findings to previously published findings of a decreased GC-induced maturation of DCs (32) may be explained by the unilateral perspective of an *in-vitro* study while our *in-vivo* model considers the complexity of the endocrine and immune system in its entirety. Hence, although the communication between GCs and DCs *via* the GR is blocked, potential negative consequences might be outshined by alternative pathways.

**Figure 6:**
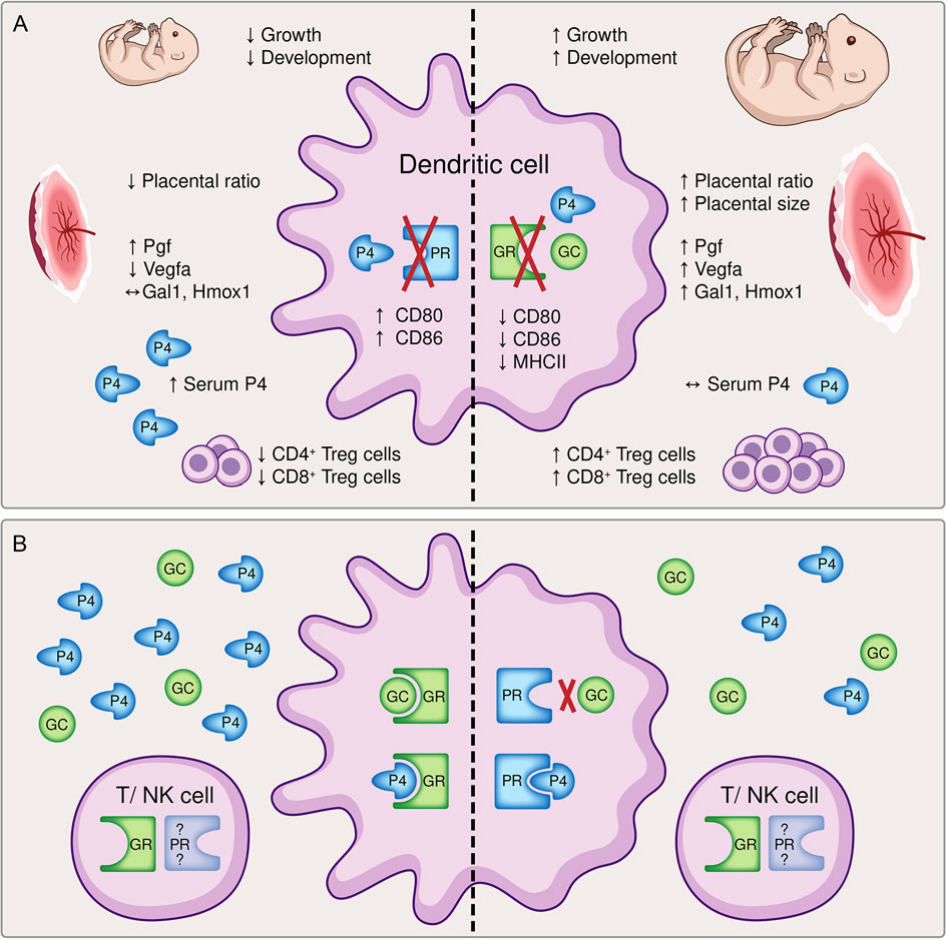
A graphical summary comparing **(A)** the effects of a dendritic cell (DC)-specific knockout of the progesterone receptor (PR, left) and the glucocorticoid receptor (GR, right) and **(B)** potential compensatory pathways available to the immune system. **(A)** The inability to respond to either progesterone (P4) or glucocorticoids (GC) resulted in opposite effects regarding fetal growth and development, placental histomorphology and function, the immune cell phenotype of DCs and the generation of pregnancy-protective CD4^+^ and CD8^+^ regulatory T cells, respectively. Left: The conditional knockout of the PR on DCs led to a significantly reduced fetal weight and histomorphological and functional impairment of the placenta. Immune adaptational processes were negatively affected as observed by an increased frequency of co-stimulatory molecules CD80/86 on CD11c^+^ DCs along with reduced frequencies of CD4^+^ FoxP3^+^ and CD8^+^ CD122^+^ regulatory T (Treg) cells, although serum progesterone concentrations were elevated (6) Right: In contrast, the conditional knockout of the GR on DCs improved fetal growth and placenta function. An augmented tolerant immune microenvironment was demonstrated by decreased expression of co-stimulatory molecules major histocompatibility complex (MHC) II and CD80/86 on CD11c^+^ dendritic cells and increased frequencies of CD4^+^ and CD8^+^ Treg cells despite similar serum progesterone levels. **(B)** The inability of progesterone or glucocorticoids to exert their function *via* the preferred receptor may foster the utilization of compensatory pathways. Left: A dysfunctional PR can be partly compensated by progesterone and glucocorticoids acting *via* the GR. Right: The binding affinity of glucocorticoids to the PR is rather low, but a disrupted GR may be counterbalanced by progesterone binding to the PR. Additionally, hormonal signaling directly *via* T cells and natural killer (NK) cells may be feasible, although the expression of the PR on T cells and NK cells is still debatable. Pgf, placental growth factor; Vegfa, vascular endothelial growth factor A; Gal1, galectin-1; Hmox1, heme oxygenase 1.

In order to evaluate the impact of steroids on DC function during pregnancy, we previously investigated the consequences of an impaired progesterone responsiveness of DCs during pregnancy, taking advantage of the selective knockout of the PR on CD11c^+^ DCs in mice (6). Interestingly, almost all effects we observed in this previous study regarding fetal growth, placenta formation and maternal immune adaptation represented to be the direct opposite to the findings of our present study, as depicted in **Figure 6A**. Initially it may be confusing that two steroid hormones, which exhibit the same immunomodulatory phenotype towards immune tolerance, show opposite effects when they are depleted individually. Hereby, receptor availability and affinity might play a role. While the GR is almost ubiquitously expressed on all immune cells (9), the expression of the PR on T cells and NK cells is still not conclusively clarified (33, 34). Hence, while a hormone mediated immune response *via* the GR might be utilized with T cell and NK cell, that alternative option may be limited for the PR. On the other hand, GC exert their function mainly *via* binding to the GR with a relative binding affinity (RBA) of 100% for dexamethasone or 85% for corticosterone (35), but alternative receptors such as membrane progestin receptors (mPR), progesterone receptor membrane components (PGRMC) or even the PR [RBA of 0.2% for Dexamethasone (36) and 2.6% for corticosterone (35)] only play a minor role (37). In contrast, progesterone can bind with highest affinity to the PR (RBA 100%), but also to the GR (RBA between 1-6% (38) or 40% (36) and the mPR and PGRMC (33, 34). Another signaling pathway of progesterone and GC is effected by means of the mineralocorticoid receptor and both hormones show similar RBAs of 22% for progesterone, 21% for dexamethasone and 18% for corticosterone in comparison to aldosterone (35). Consequently, it seems that progesterone has more options to ensure its function than GCs. The expression of mPR and PGRMC on DCs has not been clarified yet. Considering these receptors might not be expressed on DCs, the negative effect of an impaired progesterone-responsiveness of DCs on fetal development could be explained.

Another explanation may be indirect compensatory mechanisms and the efficiency of progesterone and GC action on DCs. A disrupted progesterone-DC cross talk *via* the PR cannot be compensated by GCs and progesterone acting *via* the GR (**Figure 6B**). Hence, although GCs exhibit immunomodulatory function towards immune tolerance, it might not be as efficient as progesterone. In addition, the binding affinity of progesterone to the GR may be too low to compensate for the missing PR, although increased serum progesterone level have been observed (6). As a result, the expression of co-stimulatory molecules on DCs is not suppressed leading to a reduced generation of regulatory T cells which negatively affects placental vascularization and fetal development.

In contrast, a disrupted GC-DC cross talk *via* the GR may be overcompensated by progesterone acting *via* the PR (**Figure 6B**). This would strengthen the significance of our previous findings (6) and highlights that progesterone-DC cross talk is crucial for the induction of a tolerogenic DC phenotype (6, 39, 40). In this context, also the previously described Gal-1-mediated induction of tolerogenic DCs (2), which subsequently leads to CD4^+^ Treg expansion, may be primarily progesterone dependent as placental expression of *Lgals-1* is increased in GRnegCD11c mice. Further, placental Hmox1 expression does not seem to be exclusively dependent on progesterone or the PR, respectively, as suggested by our previous findings (15), but may be specifically enabled by a functional progesterone-DC cross talk. Consequently, increased placental Hmox1 expression observed in GRnegCD11c mice might induced CD8^+^ CD122^+^ Treg cell generation supporting fetal development.

Concurrently, also placental formation and functionality was improved in GRnegCD11c mice which could also be explained by increased placental Hmox1 expression (15). Overall placental size is increased on gd 13.5, equally distributed between labyrinth and junctional zone. In contrast, towards the end of pregnancy there is a shift towards the labyrinth at the expense of the junctional zone, but equal overall placenta size. Since the placenta is not fully developed before gd 14.5 (41), differences in placental size may result from different rates of placental development due to hormone-mediated signaling (**Figure 6**). Subsequently, extensively branched villi in the labyrinth are formed by the trophoblast and fetal vasculature until birth (42) to further supports fetal supply with nutrients and oxygen while coincidently the impact of hormone production in the junctional zone may be receding in importance.

Interestingly, in both conditional KO mice, GRnegCD11c and PRnegCD11c, we observed an increased placental expression of Pgf, suggesting that progesterone as well as GCs are able to upregulate PGF to promote placental angiogenesis, especially low resistance vascular network during mid- to late pregnancy (43). In contrast, VEGF, another endothelium-specific molecule supporting placental vasculogenesis by initiating the branching of blood vessels, was only found to be upregulated in GRnegCD11c mice and downregulated in PRnegCD11c mice, suggesting the necessity of a progesterone-DC mediated induction of placental Vegfa expression.

GCs are critically required to ensure reproductive success (7). Hereby, levels of GCs need to be tightly regulated. Sufficient levels are necessary to meet the increasing energy demands throughout pregnancy (8) hereby avoiding fetal growth impairment. Further, GCs are needed for fetal organ maturation, e.g. the lung (44) and even clinically utilized to improve intra-uterine environment before conception (45) and to improve neonatal outcome by threatening preterm labor (46). However, on the other hand, excess levels of GCs, e.g. due to maternal stress perception during pregnancy, lead to adverse fetal outcome such as intra-uterine growth restriction (8) and dysfunction of the cardiovascular, metabolic, endocrine, nervous, and reproductive systems later in life of the offspring (47). Further, progesterone levels are reduced by prenatal stress which may account for the attenuation of the maternal immune system to establish fetal tolerance (15). Hence, the interaction of GCs and progesterone in a cell-specific manner and their involvement in generating immune tolerance towards the fetus needs further investigation. It would be too easy to conclude that a disrupted communication between progesterone and DCs is detrimental and *vice versa* the knockout of the GR on DCs would be beneficial for pregnancy. In order to further elucidate the mechanisms of a DC-mediated adaptive immune response during pregnancy, a PR and GR double knockout on dendritic cells with subsequent specific cell transfers might facilitate such investigations in mice. Recently, we already proposed that a tight equilibrium between progesterone and GCs may be critically required to ensure a tolerogenic immune profile to promote placental vascularization and healthy fetal growth while a disequilibrium might lead to an altered intrauterine immune profile and subsequently cause placental insufficiency and pregnancy complication (37). This notion is now underpinned by scientific data presented here and in our previous paper (6).

In summary, we successfully generated a DC-specific knockout of the GR in mice by utilizing the cre/flox system. We could demonstrate that an impaired GC-responsiveness of DCs facilitates the generation of pregnancy-protective CD4^+^ and CD8^+^ Treg cells, leading to improved placentation and fetal development. However, these effects may be mediated by compensatory mechanisms of progesterone acting *via* the PR. The potential role of GCs for the induction of immune tolerance during pregnancy needs further clarification, which also includes the impact of estrogens which exhibits antagonistic features in the uterus (48) and the impact of the mineralocorticoid receptors (49). The identification of underlying mechanisms of tissue-specific effects of GCs may also improve selective glucocorticoid therapies during pregnancy.

## Data Availability Statement

The original contributions presented in the study are included in the article/**Supplementary Material**. Further inquiries can be directed to the corresponding author.

## Ethics Statement

The animal study was reviewed and approved by the State Authority of Hamburg (ORG_952).

## Author Contributions

Conceptualization, KT and PA. Methodology, KT and LD. Investigation, KT, LD, AH, and AW. Resources, KT and PA. Writing – Original Draft, KT and PA. Comments on manuscript, all authors. All authors contributed to the article and approved the submitted version.

## Funding

This work was supported by research grants provided by the German Research Foundation to KT (TH 2126/1-1) and PCA (KFO296, AR232/25-2 and AR232/27-1) and by the State Research Funding – FV45, Authority for Science, Research and Equality, Hanseatic City of Hamburg, Germany to PCA.

## Conflict of Interest

The authors declare that the research was conducted in the absence of any commercial or financial relationships that could be construed as a potential conflict of interest.

## Publisher’s Note

All claims expressed in this article are solely those of the authors and do not necessarily represent those of their affiliated organizations, or those of the publisher, the editors and the reviewers. Any product that may be evaluated in this article, or claim that may be made by its manufacturer, is not guaranteed or endorsed by the publisher.
