# Supplementary material for: Disruption of Glucocorticoid Action on CD11c+ Dendritic Cells Favors the Generation of CD4+ Regulatory T Cells and Improves Fetal Development in Mice
Source: Front Immunol. 2021 Oct 26;12:729742. doi: 10.3389/fimmu.2021.729742 (PMC8576435; doi:10.3389/fimmu.2021.729742)
Supplement: Supplementary file 1 [file DataSheet_1.pdf]

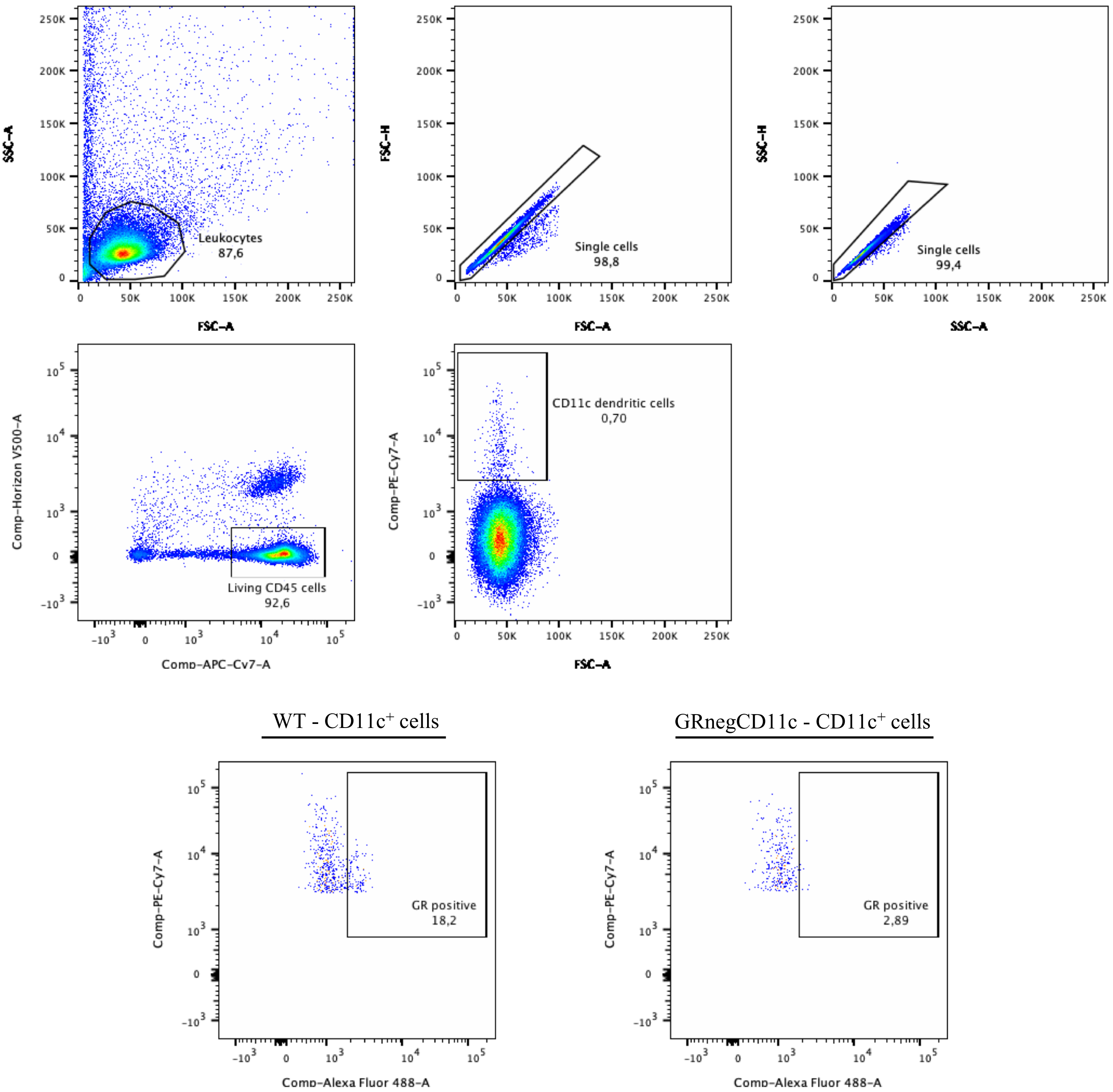

Suppl. Fig. 1: Gating stategy for the confirmation of the GR knockout on CD11c+ cells: Spleen cells from WT and GRnegCD11c mice were stimulated with 10^{-6} M progesterone or corticosterone or only medium for 15 minutes, respectively. Subsequently, samples were blocked and stained with an eFluor 506 viability dye, CD45 and CD11c . Finally, cells were fixed and permeabilized to perform intracellular staining with an anti-GR antibody.

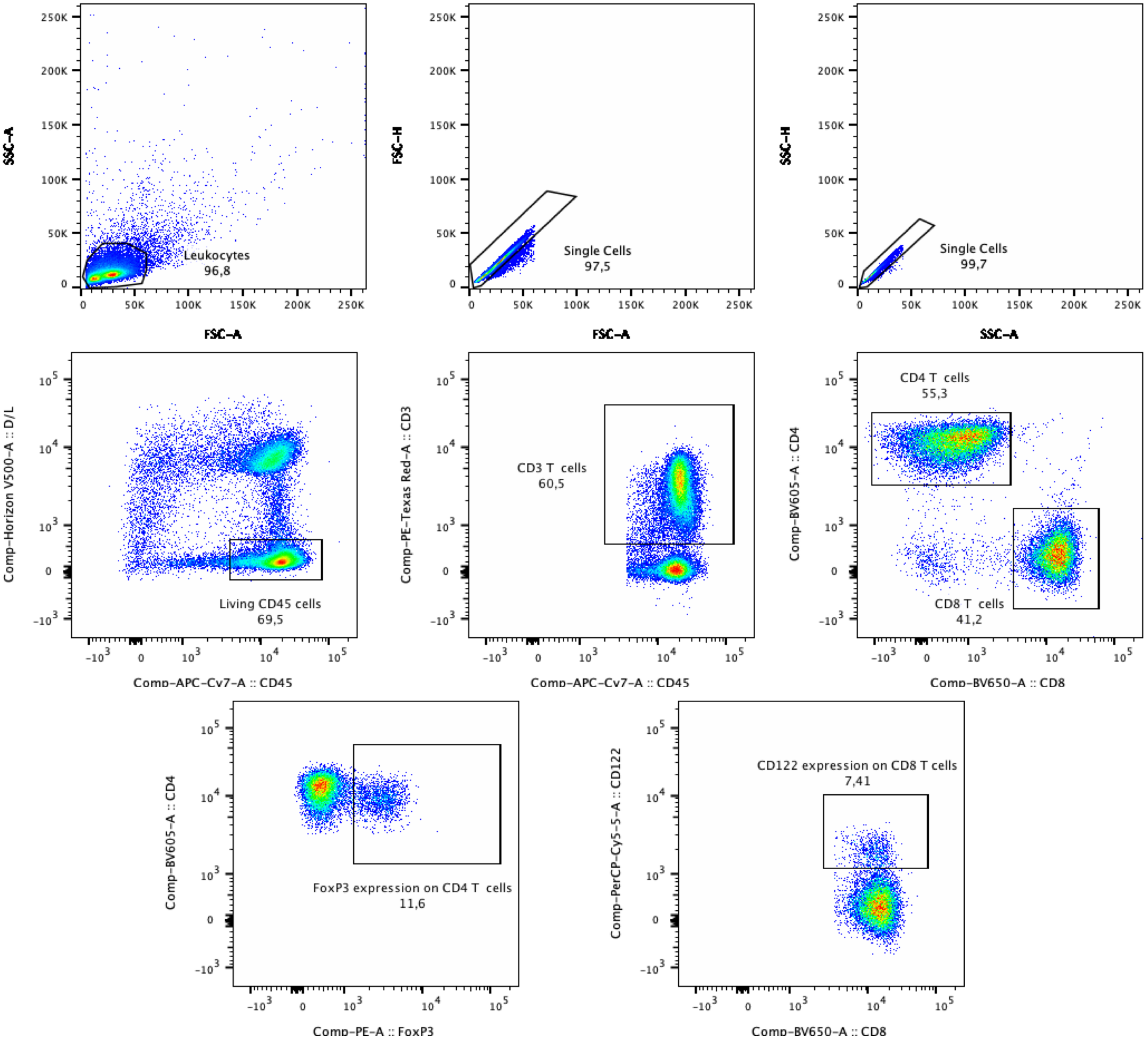

Suppl. Fig. 2: Gating stratey for the analysis of T cell subpopulations: Cells from WT and GRnegCD11c mice were blocked and stained with an eFluor 506 viability dye, CD45, CD3, CD4, CD8 and CD122. Finally, cells were fixed and permeabilized to perform intracellular staining with an anti-FoxP3 antibody.

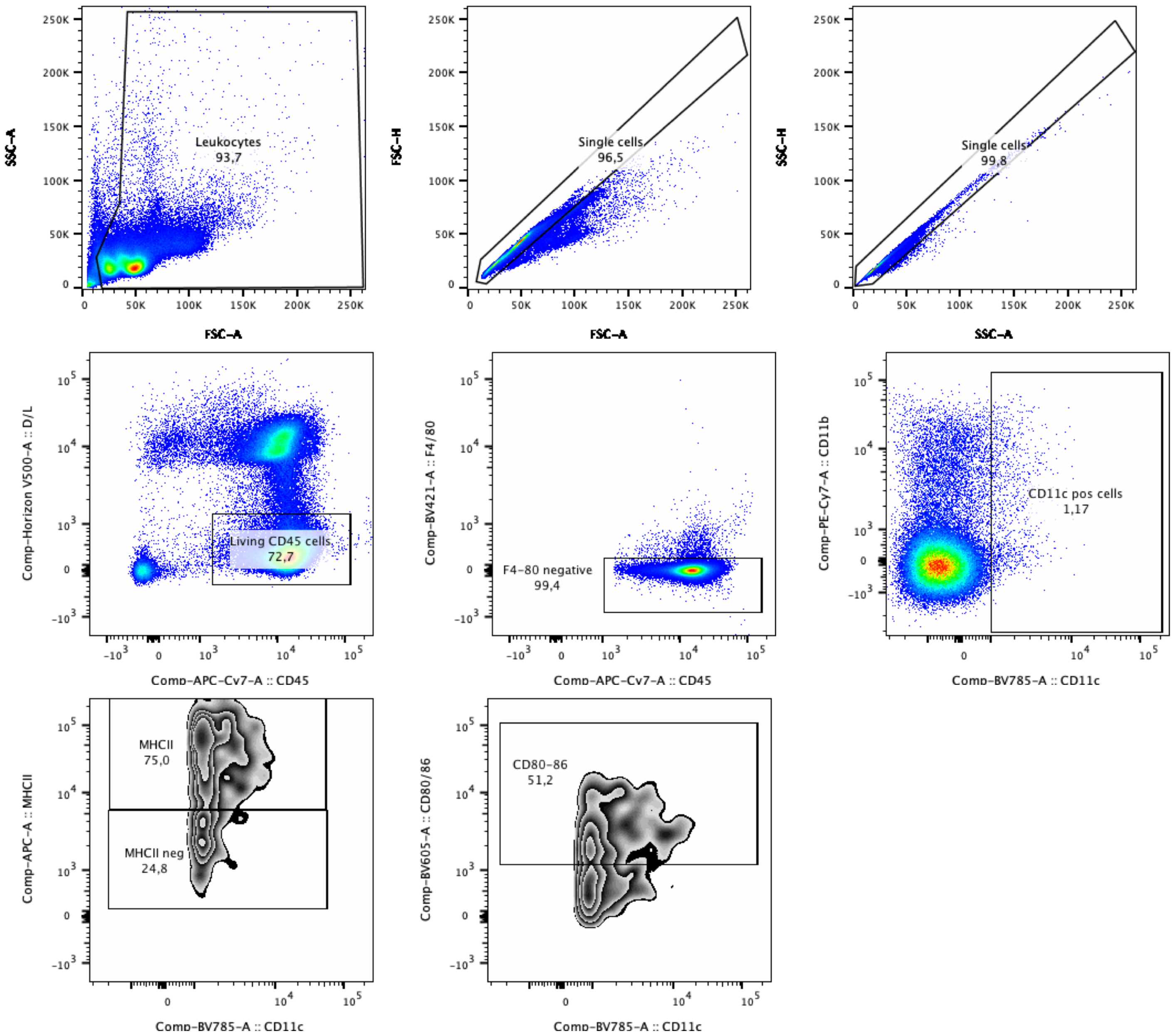

Suppl. Fig. 3: Gating strategy for the analysis of dendritic cell maturation: Cells from WT and GRnegCD11c mice were blocked and stained with an eFluor 506 viability dye, CD45, F4/80 CD11b, CD11c, MHCII and CD80/86.
